# Trends, patterns and psychological influences on COVID-19 vaccination intention: Findings from a large prospective community cohort study in England and Wales (Virus Watch)

**DOI:** 10.1016/j.vaccine.2021.09.066

**Published:** 2021-11-26

**Authors:** Thomas Byrne, Parth Patel, Madhumita Shrotri, Sarah Beale, Susan Michie, Jabeer Butt, Nicky Hawkins, Pia Hardelid, Alison Rodger, Anna Aryee, Isobel Braithwaite, Wing Lam Erica Fong, Ellen Fragaszy, Cyril Geismar, Jana Kovar, Annalan M.D. Navaratnam, Vincent Nguyen, Andrew Hayward, Robert W Aldridge, on behalf of Virus Watch Collaborative, Linda Wijlaar, Eleni Nastouli, Moira Spyer, Ben Killingley, Ingemar Cox, Vasileios Lampos, Rachel A. McKendry, Tao Cheng, Yunzhe Liu, Anne M. Johnson, Jo Gibbs, Richard Gilson

**Affiliations:** f Department of Population, Policy and Practice, UCL Great Ormond Street Institute of Child Health, 30 Guilford St, London WC1N 1EH, UK; j Francis Crick Institute, 1 Midland Rd, London NW1 1AT, UK; k Health Protection and Influenza Research Group, Division of Epidemiology and Public Health, University of Nottingham School of Medicine, Nottingham, United Kingdom; l University College London Hospital, Euston Rd, London WC1H 8NJ, United Kingdom; m Department of Computer Science, University College London, 17-19 Gordon Street, London WC1H 0AH, UK; n London Centre for Nanotechnology and Division of Medicine, London, UCL, UK; o SpaceTimeLab, Department of Civil, Environmental and Geomatic Engineering, University College London, London, UK; g Institute for Global Health, University College London, 30 Guilford St, London WC1N 1EH, UK; a Centre for Public Health Data Science, Institute of Health Informatics, University College, 222 Euston Rd, London NW1 2DA, UK; b Institute of Epidemiology and Health Care, University College London, 1-19 Torrington Place, London WC1E 7HB, UK; c Centre for Behaviour Change, University College London, 1-19 Torrington Place, London WC1E 7HB, UK; d Race Equality Foundation, 27 Greenwood Pl, London NW5 1LB, UK; e Independent Consultant, UK; f Department of Population, Policy and Practice, UCL Great Ormond Street Institute of Child Health, 30 Guilford St, London WC1N 1EH, UK; g Institute for Global Health, University College London, 30 Guilford St, London WC1N 1EH, UK; h Royal Free London NHS Foundation Trust, Pond Street, London, NW3 2QG, UK; i Department of Infectious Disease Epidemiology, London School of Hygiene and Tropical Medicine, Keppel St, London WC1E 7HT, UK

**Keywords:** COVID-19, Vaccine, Intention, Public Health

## Abstract

**Background:**

Vaccination intention is key to the success of any vaccination programme, alongside vaccine availability and access. Public intention to take a COVID-19 vaccine is high in England and Wales compared to other countries, but vaccination rate disparities between ethnic, social and age groups has led to concern.

**Methods:**

Online survey of prospective household community cohort study participants across England and Wales (Virus Watch). Vaccination intention was measured by individual participant responses to ‘Would you accept a COVID-19 vaccine if offered?’, collected in December 2020 and February 2021. Responses to a 13-item questionnaire collected in January 2021 were analysed using factor analysis to investigate psychological influences on vaccination intention.

**Results:**

Survey response rate was 56% (20,785/36,998) in December 2020 and 53% (20,590/38,727) in February 2021, with 14,880 adults reporting across both time points. In December 2020, 1,469 (10%) participants responded ‘No’ or ‘Unsure’. Of these people, 1,266 (86%) changed their mind and responded ‘Yes’ or ‘Already had a COVID-19 vaccine’ by February 2021. Vaccination intention increased across all ethnic groups and levels of social deprivation. Age was most strongly associated with vaccination intention, with 16–24-year-olds more likely to respond “Unsure” or “No” versus “Yes” than 65–74-year-olds in December 2020 (OR: 4.63, 95 %CI: 3.42, 6.27 & OR 7.17 95 %CI: 4.26, 12.07 respectively) and February 2021 (OR: 27.92 95 %CI: 13.79, 56.51 & OR 17.16 95 %CI: 4.12, 71.55). The association between ethnicity and vaccination intention weakened, but did not disappear, over time. Both vaccine- and illness-related psychological factors were shown to influence vaccination intention.

**Conclusions:**

Four in five adults (86%) who were reluctant or intending to refuse a COVID-19 vaccine in December 2020 had changed their mind in February 2021 and planned to accept, or had already accepted, a vaccine.

## Highlights

• Intention to take a COVID-19 vaccine when offered is very high in England and Wales.
• Most people who were initially reluctant to accept a COVID-19 vaccine went on to change their mind.
• Young adults and people from some minority ethnic groups are more likely to be uncertain or plan to refuse a COVID-19 vaccine.
• Both perceptions of vaccines and perceptions of COVID-19 illness severity shape vaccine intention.

## 1 Introduction

Vaccination intention, which refers to the intention to take or refuse a vaccine when offered, determines the success of any vaccination programme, alongside vaccine availability and access. In 2019, the World Health Organization listed the reluctance or refusal of vaccines as one of the top threats to global public health [1]. Patterns and drivers of vaccination intention have been shown to vary over time and region, correlating to local politics, history and religion [2].

The UK’s COVID-19 vaccination programme plans to achieve high levels of vaccination coverage across the population [3]. Procuring sufficient vaccines and delivering mainly through universal primary care has meant the UK currently has one of the highest COVID-19 vaccination rates per capita in the world [4]. Public intention to take a COVID-19 vaccine when offered is high in the UK [5], but evidence of disparities in vaccination intention between ethnic and social groups has led to significant concern among public health practitioners, the National Health Service (NHS) (which is leading the UK’s vaccine delivery), voluntary sector organisations, the media and politicians [6], [7], [8].

Previous studies have found that age, ethnicity, income and education are independently associated with COVID-19 vaccination intention [9], [10]. They found young adults, people from most minority ethnic backgrounds, people on low income and people with low education levels are more likely to be reluctant or refuse a COVID-19 vaccine [9], [10]. Reluctance or refusal to take a vaccine is contributing to disparities in COVID-19 vaccination rates, which is lower in most minority ethnic populations and in areas of higher deprivation [11], [12]. However, it is unclear to what extent disparities in COVID-19 vaccination rates are a result of differences in vaccination intention, as opposed to structural factors that determine vaccine access, such as capability to travel to a vaccination centre [13], [14].

Research conducted before the UK’s COVID-19 vaccination programme commenced found beliefs around the efficacy and safety of COVID-19 vaccines are likely to be the greatest psychological influence on vaccination intention [15]. It is not clear if this remains has remained the case since the vaccination programme began and if perceptions of the risk of COVID-19 illness also influences vaccination intention.

Disparities in COVID-19 vaccination rates are especially concerning given the greater risk of COVID-19 infection, severe illness and death in most minority ethnic populations and areas of high deprivation [16], [17]. Following concerted and targeted action to increase public intention to take a COVID-19 vaccine, there is a need to determine whether vaccination intention is changing over time.

This study aims to:

(1) Examine how COVID-19 vaccination intention has changed over time, across different populations in England and Wales.
(2) Investigate socio-demographic factors associated with current vaccination intention in England and Wales.
(3) Investigate how vaccine- and illness-related psychological factors (attitudes, beliefs and emotions) may influence vaccination intention and whether these factors vary across populations.

## 2 Methods

### 2.1 Study design and procedure

Data for this analysis were collected as part of the Virus Watch study, a large prospective household community cohort study of the transmission and burden of COVID-19 in England and Wales. The full study design and methodology has been described elsewhere [18]. Data collection using online REDCap surveys began on 24 June 2020 and is ongoing.

After enrolling in the study, an initial baseline survey collected demographic, occupation, financial and medical history data from participants. Thereafter, participants were surveyed weekly (contacted by email) on the presence or absence of symptoms that could indicate COVID-19 disease, activities undertaken prior to symptom onset, any SARS-CoV-2 swab test results, and COVID-19 vaccine uptake in the previous week. Bespoke monthly surveys collected detailed information on potential determinants of SARS-CoV-2 infection and COVID-19 disease.

### 2.2 Participants

Participants were recruited into the Virus Watch study using a range of methods including by post, social media, SMS messages or personalised letters from General Practices with tokens of appreciation for participation. Participants were eligible if all household members agreed to take part, if they had access to the internet (Wi-Fi, fixed or on a mobile phone) and an email address. At least one household member had to be able to read English to complete the surveys. Participants were not eligible if their household was larger than 6 people (due to limitations of the online survey infrastructure).

In the analyses described in this report, we excluded respondents under the age of 16 for two reasons. First, survey responses are more likely to represent parental views than views of children themselves as the surveys may be completed by an adult on their child’s behalf. Second, children were not eligible for vaccination in the UK at the time of data collection, which may have influenced parents’ intention to vaccinate their children.

### 2.3 Exposures

We explored key demographic, social, and clinical variables that could be associated with COVID-19 vaccination intention amongst adults. Age (on entry to the study) and sex (at birth) were defined *a priori* as variables of interest. Other variables of interest included self-reported ethnicity, grouped as per the following ONS categories: ‘White British’, ‘White Irish’, ‘White Other’, ‘South Asian’, ‘Other Asian’, ‘Black’, ‘Mixed’, ‘Other ethnicity’; whether born in the UK or born abroad; region of residence within England and Wales; small area-level deprivation using the Indices of Multiple Deprivation (IMD) based on postcode of residence [19]; presence of comorbidities associated with higher risk of adverse COVID-19 outcomes (as defined by Public Health England - see Appendix IA) based on data from the baseline survey; health or care worker status; and inclusion within one of the UK Joint Committee on Vaccination and Immunisation (JCVI)’s priority groups for COVID-19 vaccination (see Appendix IA) [20]. Data on exposure variables were collected through the baseline survey completed on entry into the Virus Watch study.

### 2.4 Outcomes

The primary outcome of interest was each participant’s response to the following question: *‘Would you accept a COVID-19 vaccine if offered?’*. Outcome data were collected over two separate time periods (1–14 December 2020 and 17–28 Feb 2021) through surveys sent to the whole Virus Watch cohort on the first listed day. Possible responses were: *‘Yes’, ‘No’, ‘Unsure’* and *‘Already had a COVID-19 vaccine’* (February survey only).

Between 4th-11th January 2021, psychological influences on vaccination intention were surveyed using a 13-item questionnaire measuring attitudes, beliefs and emotions related to COVID-19 illness and vaccination. This questionnaire was adapted from a 16-item measure used in the Flu Watch cohort study to measure vaccination-related attitudes during the H1N1 influenza pandemic [15]; three items were removed, and the wording of the remaining items adapted to reflect the current pandemic situation. Participants rated their agreement for all items on a 5-point Likert scale (‘strongly disagree’ – ‘strongly agree’), with negative-worded items reverse coded prior to analysis. The full questionnaire is provided in Appendix II.

### 2.5 Statistical analysis

Baseline and monthly survey response data were extracted from REDCap, linked, and analysed in Stata (version 16.0, StataCorp). Observations missing data on the primary outcome of interest were excluded from the denominators of all analyses. Respondents with missing baseline information were excluded from only the relevant explanatory variable denominator; for example, those missing a postcode were excluded from analyses by region and IMD.

To examine changes in intention over time, responses to the February survey were compared to those from December among individuals with complete data at both time points. We grouped ‘No’ and ‘Unsure’ responses due to small numbers. The percentage change in response was calculated overall and by ethnicity and IMD quintile. We suppressed low cell counts across certain categories to reduce the possibility of deductive disclosure.

We used multinomial logistic regression to derive relative odds ratios and 95% confidence intervals for the association between a range of demographic, social, and clinical participant characteristics and responses to the question ‘Would you accept a COVID-19 vaccine if offered?’ among participants who responded to both the December 2020 and February 2021 surveys. We first tested the association for each variable separately, adjusting only for age and sex (*a priori* variables). Variables that were significantly associated in univariable analyses and considered theoretically relevant to vaccine intention were included in the fully-adjusted multivariable models (“Unsure” vs “Yes” and “No” vs “Yes” in December 2020 and “Unsure” vs “Yes or Already had a vaccine” and “No” versus “Yes or Already had a vaccine” in February 2021). The multivariable models also included a random term to account for clustering at the household level.

To identify psychological influences on vaccination intention, survey responses from the January monthly survey were split into a ‘training’ dataset (*n* = 10,088 responses) for exploratory factor analysis and a ‘cross-validation’ dataset (*n =* 10,890) for confirmatory factor analysis. Further details of the approach are in Appendix III. To assess whether psychological factors differed by socio-demographic characteristics, we compared median factor scores based on their exact 95% confidence intervals and Kruskall-Wallis equality of populations rank tests by age group, sex, IMD quintile, and ethnicity among a subset of participants who responded to all three surveys (December, January and February n = 11,623).

### 2.6 Ethics

This study was approved by the Hampstead NHS Health Research Authority Ethics Committee. Ethics approval number − 20/HRA/2320.

### 2.7 Patient and public involvement

The study team worked with the Race Equality Foundation and Doctors of the World who advised on the inclusion of people from minority ethnic backgrounds in Virus Watch and set up a community advisory group to inform the ongoing design and dissemination of health equity aspects of Virus Watch. This advisory group, consisting of lay members of the public, community leaders, charities and policy experts, guided and reviewed the analyses described in this paper. Results of this and other Virus Watch analyses are disseminated to participants via the http://ucl-virus-watch.net/ website.

## 3 Results

When data were extracted on 28 February 2021, there were 22,556 households and 46,539 people taking part in Virus Watch across England and Wales, of whom 40,810 (88%) were adults aged 16 years and over and 4,858 (12%) people from minority ethnic backgrounds (Table 1).

**Table 1:** Characteristics of adult Virus Watch study participants who responded to the question on COVID-19 vaccine intention in December 2020 and again in February 2021.

	Adults responding to both surveys (n = 14,880)	
	*n*	*%*
**Sex** (at birth)		
Male	7,112	47.8
Female	7,564	50.8
Other or missing	204	1.4

**Age** (years)		
16–24	768	5.2
25–34	828	5.6
35–44	1,249	8.4
45–54	2,000	13.4
55–64	3,759	25.3
65–74	5,013	33.7
75+	1,263	8.5

**Place of birth**		
Born in UK	11,932	80.2
Born abroad	1,134	7.6
Missing	1,814	12.2

**Ethnicity**		
White British	13,220	88.8
White Irish	202	1.4
White Other	610	4.1
South Asian	251	1.7
Other Asian	100	0.7
Black	43	0.3
Mixed	143	1.0
Other ethnicity	52	0.4
Prefer not to disclose or missing	259	1.7

**Region**		
East Midlands	1,398	9.4
East of England	2,975	20.0
London	1,490	10.0
North East	729	4.9
North West	1,603	10.8
South East	2,534	17.0
South West	1,204	8.1
West Midlands	845	5.7
Yorkshire and The Humber	775	5.2
Wales	322	2.2
Missing	1,005	6.8

**IMD** (quintiles)		
1 (most deprived)	646	4.3
2	1,409	9.5
3	2,661	17.9
4	4,087	27.5
5 (least deprived)	5,072	34.1
Missing	1,005	6.8
**Health or care worker status**	558	3.8
**Priority health condition**	6,241	42.0
**JCVI priority groups**		
Group 2	926	6.2
Group 3	890	6.0
Group 4	2,849	19.2
Group 5	2,455	16.5
Group 6	2,396	16.1
Group 7	1,156	7.8
Group 8	915	6.2
Group 9	672	4.5
Not in any JCVI priority group	2,621	17.6

### 3.1 Trends in vaccination intention

Participants responded to the survey question “Would you accept a COVID-19 vaccine if offered?”. The response rate for participants aged 16 or over to the bespoke monthly survey in December 2020 was 56% (20,785/36,998) and in February 2021 was 53% (20,590/38,727), with 14,880 adults reporting across both time points. Table 1 summarises the characteristics of people who answered these surveys.

In December 2020, 18,508 (89%) participants responded “Yes”; 1,814 (9%) said they were “Unsure”; and 463 (2%) responded “No”. In February 2021, 7,847 (38%) participants responded “Already had a COVID-19 vaccine”; 12,273 (60%) responded “Yes”; 288 (1%) said they were “Unsure”; and 182 (1%) responded “No” (see Appendix IB for full description of responses by explanatory variables).

Examining only participants who answered the survey question at both timepoints, 13,411 responded “Yes” in December 2020. Of these, 13,322 (99%) went on to respond “Yes” or “Already had a COVID-19 vaccine” and 89 (1%) to respond “No” or “Unsure” in February 2021 (Table 2).

**Table 2:** Change in responses to “Would you accept a COVID-19 vaccine if offered” from December to February, by ethnicity and IMD quintile (n = 14,880).

	“Yes” in Dec 2020 N = 13,411 (90%) when asked again in Feb 2021		“No or Unsure” in Dec 2020 N = 1,469 (10%) when asked again in Feb 2021	
	Yes or Already had N = 13,322 (99%)	No or Unsure N = 89 (1%)	Yes or Already had N = 1,266 (86%)	No or Unsure N = 203 (14%)
**Age** (years)				
16–24	580 (97%)	20 (3%)	124 (74%)	44 (26%)
25–34	654 (97%)	21 (3%)	118 (77%)	35 (23%)
35–44	1,013 (99%)	11 (1%)	189 (84%)	36 (16%)
45–54	1,689 (99%)	14 (1%)	249 (84%)	48 (16%)
55–64	3,416 (100%)	15 (0%)	302 (92%)	26 (8%)
65–74	4,772 (100%)	5 (0%)	224 (95%)	12 (5%)
75+	1,198 (100%)	3 (0%)	60 (97%)	2 (3%)

**Ethnicity category (n = 12,902)**				
White British	11,972 (99%)	71 (1%)	1,028 (87%)	149 (13%)
White Irish	173 (98%)	3 (2%)	22 (85%)	4 (15%)
White Other	489 (99%)	3 (1%)	94 (80%)	24 (20%)
South Asian	214 (99%)	3 (1%)	31 (91%)	3 (9%)
Other Asian	79 (99%)	1 (1%)	14 (70%)	6 (30%)
Black	26 (96%)	1 (4%)	14 (88%)	2 (13%)
Mixed	115 (97%)	4 (3%)	19 (79%)	5 (21%)
Other Ethnicity	35 (100%)	0 (0%)	14 (82%)	3 (18%)

**IMD Quintile (n = 13,715)**				
1 (Most deprived)	548 (99%)	4 (1%)	78 (83%)	16 (17%)
2	1,187 (99%)	8 (1%)	171 (80%)	43 (20%)
3	2,371 (99%)	20 (1%)	231 (86%)	39 (14%)
4	3,691 (99%)	21 (1%)	326 (87%)	49 (13%)
5 (Least deprived)	4,640 (99%)	24 (1%)	365 (89%)	43 (11%)

**Region (n = 13,875)**				
East Midlands	1,258 (99%)	8 (1%)	118 (89%)	14 (11%)
East of England	2,671 (99%)	17 (1%)	253 (88%)	34 (12%)
London	1,250 (99%)	12 (1%)	185 (81%)	43 (19%)
North East	656 (99%)	7 (1%)	57 (86%)	9 (14%)
North West	1,414 (99%)	13 (1%)	142 (81%)	34 (19%)
South East	2,323 (100%)	6 (0%)	177 (86%)	28 (14%)
South West	1,110 (100%)	5 (0%)	84 (94%)	5 (6%)
West Midlands	762 (99%)	7 (1%)	67 (88%)	9 (12%)
Yorkshire and The Humber	702 (100%)	0 (0%)	60 (82%)	13 (18%)
Wales	291 (99%)	2 (1%)	28 (97%)	1 (3%)

Again, examining only participants who answered the survey question at both timepoints, 1,469 responded “No” or “Unsure” in December 2020. Of these, 1,266 (86%) went on to respond “Yes” or “Already had a COVID-19 vaccine” in February 2021. 203 (14%) responded “No” or “Unsure” once again in February 2021. The magnitude of this shift in intention to accept a COVID-19 vaccine was high across all ethnic groups measured ranging from 70% in people from the Other Asian ethnic background to 91% in people from South Asian ethnic backgrounds (Table 2). It was also high across all age groups (ranging from 74% in 16–24 year-olds to 97% in over 75 year-olds) IMD quintiles (ranging from 80% to 89%), and regions (ranging from 81% in London to 97% in Wales).

### 3.2 Factors associated with uncertainty or intention to refuse vaccination

**Fig. 1:**
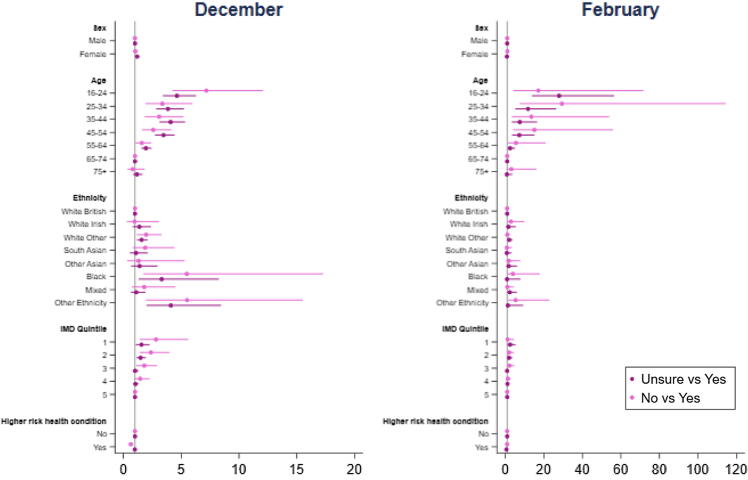
Odds ratio estimates and 95% confidence intervals from multinomial multivariable logistic regression models exploring factors associated with intention to accept a COVID-19 vaccine (‘Unsure’ vs ‘Yes’ and ‘No’ vs ‘Yes’) in December 2020 and February 2021 (see tables in Appendix IB, IC).

Sex at birth, age group, ethnicity, IMD quintile and having a priority health condition were included in all fully-adjusted multivariable regression models (Fig. 1). In the final models, age was most strongly associated with vaccination intention in both models, with intention to accept a COVID-19 vaccine inversely related to age. In December 2020, 16–24 year-olds were more likely to say Unsure and No versus ‘Yes’ compared to 65–74 year-olds (‘Unsure’: OR 4.63, 95 %CI 3.42, 6.27, p < 0.001; ‘No’: OR 7.17, 95 %CI 4.26, 12.07, p < 0.001). In February 2021, relative disparities between age groups had widened, with 16–24 year-olds much more likely to say ‘Unsure’ and ‘No’ compared to 65–74 year-olds (‘Unsure’: OR 27.92, 95 %CI 13.79, 56.51, p < 0.001; ‘No’: OR 17.16, 95 %CI 4.12, 71.55, p < 0.001).

In December 2020, females were more likely to respond ‘Unsure’ compared to males (OR 1.19, 95% CI 1.08, 1.32 p < 0.001) but the odds of responding ‘No’ were not found to be associated with sex and there was no significant difference by sex observed in February 2021. People with a health condition that was prioritised for COVID-19 vaccination (as defined by Public Health England - see Appendix IA) were less likely to respond ‘No’ compared to those without a priority health condition in December 2020 (OR 0.6495% CI 0.48, 0.87, p = 0.004).

In December 2020, ethnicity was associated with vaccine intention after adjustment for all other explanatory variables. People from White Other (‘Unsure’: OR 1.57., 95 %CI 1.18, 2.10, p = 0.002; ‘No’: OR 1.97, 95 %CI 1.17, 3.31, p = 0.011), Black (‘Unsure’: OR 3.31, 95 %CI 1.32, 8.26, p = 0.010; ‘No’: OR 5.49, 95 %CI 1.74, 17.27, p = 0.004), and Other (‘Unsure’: OR 4.10, 95 %CI 1.99, 8.46, p < 0.001; ‘No’: OR 5.51, 95 %CI 1.95, 15.52, p = 0.001) ethnic backgrounds were more likely to say ‘No’ and ‘Unsure’ versus ‘Yes’ compared with those from White British backgrounds. In February 2021, this association was weaker and disparities in vaccine intention between ethnic groups were reduced. Only people from Other ethnic backgrounds (OR 5.36, 95 %CI 1.26, 22.92, p = 0.023) remained more likely to say ‘No’ compared to White British people, and only people from White Other ethnic backgrounds (OR 2.03, 95 %CI 1.13, 3.66, p = 0.018) remained more likely to say ‘Unsure’ compared to White British people.

Across both time points, there was a gradient in vaccine intention by local area deprivation. In December 2020, people living in the two most deprived quintiles (IMD = 1 and IMD = 2) were more likely to say ‘Unsure’ and people living in the three most deprived quintiles (IMD = 1 and IMD = 2 and IMD3) were more likely to say ‘No’ compared to those living in the least deprived quintile (IMD = 5). In February 2021, those from IMD = 1 and IMD = 2 were more likely to say ‘Unsure’ (OR 2.51, 95 %CI 1.17, 5.37, p = 0.018 & OR 1.97, 95 %CI 1.07, 3.60, p = 0.028) and people from areas in IMD = 3 were also more likely to answer ‘No’ (OR 2.20, 95 %CI 1.04, 4.65, p = 0.038) compared to those living in least deprived quintile.

### 3.3 Psychological influences on vaccination intention

Exploratory factor analysis (EFA) of responses to the 13-item questionnaire suggested a two-factor structure. Factor 1 comprises beliefs and concerns around the efficacy, safety, side-effects, and time burden of vaccination and is made up of eight questions (items 6–13 in questionnaire, see supplementary materials), e.g. “I am concerned that the COVID-19 vaccine will not have been tested enough”. Factor 2 comprises risk perception and concern around acquiring, transmitting, and suffering severe effects of COVID-19 and is made up of four questions (items 1–4 in questionnaire), e.g. “I do not think that I am at risk of COVID-19”. A single item (item 5: “worry about time off work/education due to COVID-19”) was removed during EFA due to improved scale reliability after removal (Cronbach’s α 0.75 vs 0.78), and low communality and factor loadings. This item was considered theoretically relevant and retained as an individual item in later analyses. Removal of this item did not substantially affect the EFA results. All indices of relative and absolute fit based on confirmatory factor analysis (CFA) indicated that the two-factor EFA model was the best fit to the data, compared to univariable models including or excluding Item 5. Detailed EFA results pre- and post-removal and CFA results including factor loadings and indices of relative and absolute fit are reported in Appendix III.

Factors 1 and 2 were found to consistently predict responding “Unsure” or “No” versus “Yes” when participants were asked about their vaccination intention (Table 3). The first factor, relating to COVID-19 vaccines, demonstrated the strongest association with being unsure about vaccination (December OR: 0.23 [0.20, 0.26]; February OR: 0.20 [0.17, 0.24]) and intended refusal (December OR: 0.15 [0.12, 0.20]; February OR: 0.24 [0.18, 0.32]). The second factor, relating to COVID-19 illness, was also predictive of being unsure (December OR: 0.59 [0.54, 0.65]; February OR: 0.33 [0.28, 0.40]) and intended refusal (December OR: 0.38 [0.32, 0.46]; February OR: 0.45 [0.34, 0.61]). Worries about missing work or education due to COVID-19 (item 5), for which analyses were limited to participants who reported being in work or education, were associated with an increased risk of being unsure about taking a COVID-19 vaccine (OR:1.11 [1.03, 1.19]), but not intended refusal (OR: 1.09 [0.94, 1.27]) in December only.

**Table 3:** Odds Ratios between Psychological Factors and COVID-19 Vaccine Intention among participants who responded to all three surveys (n = 11,623).

	Odds Ratio [95% CI]			
	December		February	
	Unsure vs Yes	No vs Yes	Unsure vs Yes	No vs Yes
Factor 1 - Beliefs and concerns around vaccination	0.23 (0.20, 0.26)	0.15 (0.12, 0.20)	0.20 (0.17, 0.24)	0.24 (0.18, 0.32)
Factor 2 - Illness-related risk perception and concern	0.59 (0.54, 0.65)	0.38 (0.32, 0.46)	0.33 (0.28, 0.40)	0.45 (0.34, 0.61)
Item 5 - ‘I was worried about having to take time off work/education because of COVID-19′*	1.11 (1.03, 1.19)	1.09 (0.94, 1.27)	1.19 (0.99, 1.44)	1.20 (0.98, 1.47)

*limited to participants in work/education at baseline (n = 4,595).

Both psychological factors varied by age (Table 4), with more positive vaccination-related attitudes and greater concern about COVID-19 illness in older age groups, based on Factors 1 and 2 (e.g., for both factors, median for 16–24 years: 4, 95% CI: 4,4; 75 + years: 5 95% CI: 5, 5). White British participants had higher median scores (5, 95% CI: 5, 5) for Factor 1, indicating more positive views about COVID-19 vaccines compared to participants from several minority ethnic minority groups, including participants identifying as Black (4, 95 %CI: 3.5, 4), South Asian (4, 95 %CI: 4, 4.5), Other Asian (4, 95 %CI: 4, 4.5), White Other (4.5, 95 %CI: 4.5, 4.5), and Other Ethnicity (4, 95 %CI: 4, 4.93). Perceptions and concerns about COVID-19 illness did not differ by ethnicity.

**Table 4:** Median Factor Scores, 95% Confidence Intervals and Kruskal-Wallis equality of populations rank test p-values by Socio-Demographic Characteristics (n = 11,623).

		Factor 1 Beliefs and concerns around vaccination		Factor 2 Illness-related risk perception and concern		Item 5 ‘I was worried about having to take time off work/education because of COVID-19′*	
		Median [95% CI]	p-value	Median [95% CI]	p-value	Median [95% CI]	p-value
Age Group	16–24	4 (4–4)	<0.001	4 (4, 4)	<0.001	4 (3, 4)	<0.001
	25–34	4.5 (4.5–4.5)		4 (4, 4)		3 (3, 3)	
	35–44	4.5 (4, 4.5)		4 (4, 4)		3 (3, 3)	
	45–54	4.5 (4.5, 4.5)		4.5 (4.5, 4.5)		3 (3, 3)	
	55–64	5 (5, 5)		4.5 (4.5, 4.5)		3 (3, 3)	
	65–74	5 (5,5)		5 (5, 5)		2 (2, 3)	
	75+	5 (5, 5)		5 (5, 5)		2 (2, 3)	

Sex	Male	5 (4.5, 5)	0.190	4.5 (4.5, 4.5)	0.004	3 (3, 3)	0.381
	Female	5 (5, 5)		4.5 (4.5, 4.5)		3 (3, 3)	

Ethnicity	White British	5 (5, 5)	<0.001	4.5 (4.5, 4.5)	0.002	3 (3, 3)	0.128
	White Irish	5 (4.5, 5)		4.5 (4.5, 5)		3 (2, 4)	
	White Other	4.5 (4.5, 4.5)		4.5 (4.5, 4.5)		3 (3, 3)	
	South Asian	4 (4, 4.5)		4.5 (4.5, 5)		3 (3, 4)	
	Other Asian	4 (4, 4.5)		4.5 (4, 4.5)		3 (3, 4)	
	Black	4 (3.5, 4)		4.5 (4, 5)		2 (1.33,3)	
	Mixed	4.5 (4, 5)		4.5 (4, 4.5)		3 (2, 4)	
	Other Ethnicity	4 (4, 4.93)		5 (4, 5)		3 (2.13,4)	

IMD	1 (Most deprived)	4.5 (4.5, 5)	<0.001	4.5 (4.5, 4.5)	0.440	3 (3, 3)	0.009
	2	4.5 (4.5, 5)		4.5 (4.5, 4.5)		3 (3, 3)	
	3	5 (4.5, 5)		4.5 (4.5, 4.5)		3 (3, 3)	
	4	5 (4.5, 5)		4.5 (4.5, 4.5)		3 (3, 3)	
	5 (Least deprived)	5 (5, 5)		4.5 (4.5, 4.5)		3 (3, 3)	

*Limited to participants in work/education at baseline.

## 4 Discussion

In this study of over 20,000 adults from the Virus Watch cohort, the number of people who intended to accept, or had already accepted, a COVID-19 vaccine when offered increased from 89% in December 2020 to 98% in February 2021. Over four in five adults (86%) who were uncertain or intending to refuse a COVID-19 vaccine in December 2020 had changed their mind and planned on accepting, or had already accepted, a vaccine in February 2021. This shift was observed at a similar magnitude across all ethnic groups measured, all levels of social deprivation and all age groups. Despite this shift, disparities in vaccine intention still exist. Young adults and people from White Other and Other ethnic backgrounds were more likely to intend to refuse or be unsure about taking a COVID-19 vaccine relative to older adults and White British people respectively.

Concerns about COVID-19 vaccines and concerns about COVID-19 illness predicted intention to take a vaccine. Older adults were the least likely to have concerns about the vaccine and most likely to have concerns about COVID-19. White British people had fewer concerns about COVID-19 vaccines than people from most minority ethnic groups, but there were no differences between ethnic groups regarding concerns about contracting and/or becoming unwell with COVID-19 illness.

Our cohort analysis of trends in vaccine intention provides evidence that many individuals have changed their minds to become pro-vaccine. This supports cross-sectional surveys that have found COVID-19 vaccination intention has increased over time in the UK [5], [21], [22]. Previous studies have found age, ethnicity and social deprivation are independently associated with intention to take a COVID-19 vaccine [9], [10]. This study confirms that younger adults are more likely to be unsure or intend to refuse a COVID-19 vaccine compared to older adults. We find the association between ethnicity and vaccine intention has weakened. In February 2021, only people from Other ethnic backgrounds were more likely to refuse, and only people from White Other ethnic groups were more likely to be unsure about taking a COVID-19 vaccine compared to White British people. This is a substantial change from December 2021 and differs from existing evidence on ethnic disparities in COVID-19 vaccine intention [9], [10]. Similarly, the relationship between deprivation and vaccine intention (where more deprived groups were associated with lower levels of intention to be vaccinated) has weakened but not disappeared.

It is possible that public health communications to promote vaccination uptake and participatory community engagement, such as using places of worship as vaccination centres, have contributed to this shift. It is also possible that growing numbers of people being vaccinated during the period of this study has contributed to the change in intention through network effects and anticipated regret. It is especially encouraging that the intention to accept a COVID-19 vaccine has increased across all age, ethnic and social deprivation groups. Repeating offers of a COVID-19 vaccine to people may be important as large numbers of people have changed their minds over the course of a few months. Our findings also suggest that communications focusing on COVID-19 vaccine safety and effectiveness may be more effective than those focusing on COVID-19 illness risk and perception.

Despite the pro-vaccine shift observed and the narrowing of ethnic disparities in vaccine intention, disparities in vaccination rates between ethnic groups remain wide [23]. It suggests differences in vaccine intention may not be the only cause of ethnic disparities in vaccination rates and that barriers to vaccine access may also be contributing. A recent analysis of survey data from 1.4 million adults aged over 55 found people from most minority ethnic backgrounds were more likely to report difficulty walking and difficulty performing usual activities compared to White British people [24]. The Department for Transport’s national statistics from 2019 shows 39 per cent of Black adults live in households without a car compared to 17 per cent of White adults [25]. An analysis of survey data from the UK Household Longitudinal Survey found Black adults and Asian adults over the age of 65 are both around twice as likely to report having no close friends and no friends who live locally [26]. Each of these are potential structural barriers to vaccine access that may be contributing to lower vaccination rates in minority ethnic groups. Door-to-door vaccines, which have been piloted in some parts of the UK, may be effective at increasing vaccination rates and reducing ethnic disparities.

In our separate analysis of psychological influences on vaccine intention, beliefs and concerns about COVID-19 vaccines were strongly associated with intended vaccine uptake. This is in keeping with research conducted prior to the UK COVID-19 vaccination programme commencing which found beliefs around the efficacy, development, risks and importance of COVID-19 vaccines strongly predicted intention to accept a vaccine [15]. This study also finds beliefs and concerns around COVID-19 illness consistently predict vaccination intention. Our analysis was not able to consider structural reasons that determine vaccination intention beyond the role of missing work and education. Specifically, we did not measure conspiratorial beliefs and views of healthcare and medicine or trust in the government which have been previously found to affect vaccination intention [27]. We also did not collect data on how vaccine intention was affected by vaccine characteristics (including brand of vaccine, protection duration, location of manufacture and approval status), which have been shown to be associated with vaccine acceptance [28], [29].

### 4.1 Strengths and limitations

Virus Watch is a national household community cohort study. Individuals in the study were geographically distributed across England and Wales and the cohort was diverse in terms of age, sex, ethnicity, and socio-economic composition. This is a large cohort study of vaccination intention in England and Wales and has a large number of participants from minority ethnic backgrounds. However, given participation in the Virus Watch study is voluntary and sampling non-random, the cohort is likely biased toward people concerned about COVID-19 and participants were more likely to be White British, over the age of 65 and have a higher income than the general population. As a result, the data presented in this paper likely overestimate the degree of vaccine intention in England and Wales between December 2020 and February 2021 as people from minority ethnic backgrounds, younger people and those living in more deprived areas are more likely to be vaccine hesitant, due to structural differences in access to and participation in healthcare and medical research [30], [31], [32]. Our multivariable regression analyses adjust for age, ethnicity and IMD which should reduce this bias, however, any bias resulting from residual confounding means that our results are likely overestimates of the true magnitude of the association.

Despite the cohort size, samples were too small to disaggregate ethnic groups into more granular categories. Guided by our community advisory board, participants who expressed uncertainty were separated from participants intending to refuse COVID-19 vaccines in our analysis of factors associated with vaccine intention, as it was felt that these groups characterise different populations. Sample sizes precluded this separation in our analyses of temporal trends and psychological influences on vaccine intention. Another important limitation is that only households with at least one member able to read English language and access mobile or broadband internet were able to take part in the study. Guided by our community advisory board, Virus Watch study surveys have since been translated into 9 languages to allow non-English speakers to participate.

### 4.2 Conclusions

The considerable increase in intention to accept a COVID-19 vaccine across all ethnic and social groups when offered observed in this study is encouraging.

## Funding

The research costs for the study have been supported by the MRC Grant Ref: MC_PC 19,070 awarded to UCL on 30 March 2020 and MRC Grant Ref: MR/V028375/1 awarded on 17 August 2020. The study also received $15,000 of Facebook advertising credit to support a pilot social media recruitment campaign on 18th August 2020. This study was supported by the Wellcome Trust through a Wellcome Clinical Research Career Development Fellowship to RA [206602]. The sponsors of the study had no role in conducting this analysis or drafting this manuscript.

## Data Availability

We aim to share aggregate data from this project on our website and via a “Findings so far” section on our website - https://ucl-virus-watch.net/. We will also be sharing individual record level data on a research data sharing service such as the Office of National Statistics Secure Research Service. In sharing the data we will work within the principles set out in the UKRI Guidance on best practice in the management of research data. Access to use of the data whilst research is being conducted will be managed by the Chief Investigators (ACH and RWA) in accordance with the principles set out in the UKRI guidance on best practice in the management of research data. We will put analysis code on publicly available repositories to enable their reuse.

## Declaration of Competing Interest

The authors declare the following financial interests/personal relationships which may be considered as potential competing interests: Robert Aldridge reports financial support was provided by Medical Research Council. Robert Aldridge reports financial support was provided by Facebook Inc. Robert Aldridge reports financial support was provided by Wellcome Trust. The research costs for the study have been supported by the MRC Grant Ref: MC_PC 19070 awarded to UCL on 30 March 2020 and MRC Grant Ref: MR/V028375/1 awarded on 17 August 2020. The study also received $15,000 of Facebook advertising credit to support a pilot social media recruitment campaign on 18th August 2020. This study was supported by the Wellcome Trust through a Wellcome Clinical Research Career Development Fellowship to RA [206602]. The sponsors of the study had no role in conducting this analysis or drafting this manuscript. A.H serves on the UK New and Emerging Respiratory Virus Threats Advisory Group. S.M serves on the Scientific Advisory Group for Emergencies. J.B is Chief Executive of Race Equality Foundation. N.H is and independent consultant advising the Virus Watch study team on framing of results. A.M.J was a Governor of Wellcome Trust from 2011-18 and is Chair of the Committee for Strategic Coordination for Health of the Public Research.
